# A Low FODMAP Diet Supplemented with L-Tryptophan Reduces the Symptoms of Functional Constipation in Elderly Patients

**DOI:** 10.3390/nu16071027

**Published:** 2024-04-01

**Authors:** Cezary Chojnacki, Marta Mędrek-Socha, Aleksandra Błońska, Janusz Błasiak, Tomasz Popławski, Jan Chojnacki, Anita Gąsiorowska

**Affiliations:** 1 Department of Clinical Nutrition and Gastroenterological Diagnostics, Medical University of Lodz, 90-647 Lodz, Poland; marta.medrek-socha@umed.lodz.pl (M.M.-S.); aleksandra.blonska@umed.lodz.pl (A.B.); jan.chojnacki@umed.lodz.pl (J.C.); 2 Faculty of Medicine, Collegium Medicum, Mazovian Academy in Plock, 09-402 Plock, Poland; j.blasiak@mazowiecka.edu.pl; 3 Department of Pharmaceutical Microbiology and Biochemistry, Medical University of Lodz, 90-236 Lodz, Poland; tomasz.poplawski@umed.lodz.pl; 4 Department of Gastroenterology, Medical University of Lodz, 92-213 Lodz, Poland; anita.gasiorowska@umed.lodz.pl

**Keywords:** functional constipation, L-tryptophan, 5-hydroxyindoleacetic acid, kynurenine, indican

## Abstract

(1) Background: The elderly suffer from functional constipation (FC), whose causes are not fully known, but nutritional factors may play a role. The aim of the present study was to assess the effect of a low FODMAP diet supplemented with L-tryptophan (TRP) on its metabolism and symptoms of functional constipation in elderly patients. (2) Methods: This study included 40 people without abdominal complaints (Group I, controls) and 60 patients with FC, diagnosed according to the Rome IV Criteria (Group II). Two groups were randomly selected: Group IIA (*n* = 30) was qualified for administration of the low FODMAP diet, and the diet of patients of Group IIB (*n* = 30) was supplemented with 1000 mg TRP per day. The severity of abdominal symptoms was assessed with an abdominal pain index ranging from 1 to 7 points (S-score). The concentration of TRP and its metabolites, 5-hydroxyindoleacetic acid (5-HIAA), kynurenine (KYN), and 3-indoxyl sulfate (3-IS) in urine were determined using the LC-MS/MS method. (3) Results: In Group II, 5-HIAA concentration in urine was lower, and KYN and 3-IS concentrations were higher than in the control group. A negative correlation was found between the S-score and urinary concentration of 5-HIAA (*p* < 0.001), and 3-IS concentration was positively correlated with the S-score. However, the correlation between the S-score and 3-IS concentration was negative *(p* < 0.01). After a dietary intervention, 5-HIAA concentration increased in both groups, and the severity of symptoms decreased, but the decrease was more pronounced in Group IIB. (4) Conclusion: A low FODMAP diet supplemented with L-tryptophan has beneficial effects in elderly patients suffering from functional constipation.

## 1. Introduction

The worldwide prevalence of chronic constipation is estimated to be 12% to 19% in different populations [1]. However, 15–30% of people over 65 years of age are diagnosed with functional constipation [2]. The Rome IV Criteria distinguishes constipation-predominant irritable bowel syndrome (IBS-C) and functional constipation (chronic idiopathic constipation, FC, CIC) [3,4]. Criteria of both syndromes require two or more of the following symptoms above 25% of the time: straining, lumpy or hard stools, the sensation of anorectal obstruction, manual stool evacuation, and fewer than three spontaneous bowel movements per week for the last 3 months, as well as onset at least 6 months before diagnosis. The symptom differentiating IBS-C and FC is abdominal pain, which rarely occurs in FC and is usually not related to defecation [5]. The causes of FC are multifactorial, including psychological disorders, colonic dysmotility, changes in the gut microbiome, nutritional factors, and others [6,7]. Furthermore, in the aging process, various involute changes occur in the body, including the digestive tract [8,9]. These processes also include the release of hormones and neurotransmitters, which regulate the motor and secreting activity of the intestines [10]. The changes also include disturbance of serotonin (5-HT), which is related to functional diseases of the gastrointestinal tract (GIT) [11,12,13,14]. The substrate for 5-HT synthesis is L-tryptophan (TRP), which is provided exclusively with the diet. More than 90% of TRP is metabolized in the GIT in the serotonin (5-HT), kynurenine, and indole pathways, in the proportion of approximately 2%, 95%, and 3%, respectively [15]. Enzymes that initiate the conversion of TRP in these pathways, tryptophan hydroxylase 1 (TPH-1), indoleamine 2,3-dioxygenase (IDO-1), and bacterial tryptophanase (TNA), compete for access to TRP [16]. These metabolic pathways are balanced in healthy people, but this balance can be altered by many factors, including tryptophan intake and intestinal microbiome [17]. Metabolism of tryptophan may be the key to correct diagnosis and effective treatment of functional gastrointestinal diseases [18,19]. Numerous tryptophan metabolites influence the functioning of the gastrointestinal tract in different ways. For example, 5-HT, through various receptors, can directly stimulate motor activity or have a relaxing effect. On the other hand, kynurenines mainly influence the nervous system’s function. Some of them have neuroprotective properties, but others may have neurotoxic effects. A deficiency of 5-HT or an increase in kynurenine metabolites may cause mental mood disorders and accompanying dysfunction of the GIT. The coexistence of these diseases is especially common in the elderly. The metabolites of the indole pathway mainly have anti-inflammatory and immune-regulatory properties but also influence functions of the gut–brain axis. These complex mechanisms of the impact of TRP metabolites on the digestive tract are still not fully understood, which justifies further research. We have previously shown that serum 5-HT levels and urinary excretion of 5-HIAA in patients with diarrhea-predominant IBS are higher than in healthy individuals and IBS-C patients [20]. In another study, we showed that limiting the intake of TRP had a beneficial effect on IBS-D patients treated with a low FODMAP diet [21]. In these studies, only metabolites of the 5-HT pathway were assessed. In treating chronic constipation, in addition to an appropriate diet, various pharmacological drugs are used, such as laxatives, pro-kinetics, enhancing intestinal secretion, inhibitors of bile acid transporters and ion exchangers, and others. There is no available literature regarding TRP supplementation in treating this syndrome. The present study aimed to assess the effect of a low FODMAP diet with supplementation of TRP on symptoms of functional constipation in elderly patients concerning the level of selected metabolites of all pathways of TRP metabolism.

## 2. Material and Methods

### 2.1. Participants

This study included 40 subjects without any abdominal complaints (Group I, controls) and 60 patients with functional constipation (Group II, FC) aged 66–84 years. Patients were diagnosed according to the Rome IV Criteria [3], taking into account the symptoms detailed in the previous section, including an occurrence of more than 25% of the time of the following: straining, lumpy, or hard stools, the sensation of incomplete evacuation or anorectal obstruction, manual support to facilitate defecation, and fewer than three spontaneous bowel movements per week. The severity of abdominal symptoms was assessed and scored between 1 and 7 points and was based on patient notes in the diary daily for a month before and during treatment. After completing the diagnostic tests, two subgroups of patients were randomly selected. Group IIA was qualified for treatment with the low FODMAP diet. Group IIB was on a tryptophan supplementation diet, and a dose of 1000 mg per day was added to the low FODM. All patients underwent endoscopic and histological examination of colonic mucosa to rule out organic prominent intestine diseases. The inclusion criteria for the nutritional intervention were patients whose intensity of abdominal symptoms was above 21 points in total. Among this group, there were three patients with mild hypertension, four with poorer glucose tolerance, and four with single colon diverticula. The exclusion criteria were the presence of organic diseases of the gastrointestinal tract, allergy and IgG-dependent food intolerance, allergy and food intolerance, small intestinal bacterial overgrowth, liver and renal diseases, diabetes, dominant symptoms of anxiety or depression, and the use of antibiotics, probiotics, antipsychotic, and antispasmodic drugs at least one month before enrolling in this study.

### 2.2. Laboratory Tests

The laboratory tests included hemoglobin, blood cell count, the concentration of C-reactive protein, glucose, glycated hemoglobin, lipids, bilirubin, iron, urea, creatinine, the glomerular filtration rate, thyroid hormone antibodies to tissue transglutaminase, and the activity of AST, ALT, GGTP, and AP, as well as amylase and lipase, total serum IgE and IgG concentration, and deaminated gliadin peptide.

The concentration of C-reactive protein (CRP) was determined by a photometric assay in COBAS INTEGRA 800 (Roche Diagnostic, Basel, Switzerland). Fecal calprotectin (FC) was evaluated using a sandwich ELISA test in a Quantum Blue Reader (Buhlmann Diagnostics, Amherst, NH, USA). In the morning, urine samples for TRP and its metabolites testing were collected on an empty stomach into a 0.1% hydrochloric acid solution as a stabilizer. We determined the concentration of TRP and its following metabolites in urine: 5-hydroxy indole acetic acid (5-HIAA), kynurenine (KYN), and 3-indoxyl sulfate (Indican) using liquid chromatography with tandem mass spectrometry (LC–MS/MS in accordance with the manufacturer’s instructions (Ganzimmun Diagnostics AG, Mainz, Germany; D-ML-13147-01-01, accepted by the European Parliament—No. 765/2008). The levels of these metabolites were expressed in mg per gram of creatinine (mg/gCr). 5-HIAA, KYN, and 3-IS were treated as indicators of TRP metabolism’s 5-HT, kynurenine, and indole pathways. All laboratory materials were collected on the same day.

### 2.3. Nutritional Intervention

Patients were recommended to record the type and quantity of products consumed daily for 30 days before investigations in the nutritional diary. The nutritional calculator of the Kcalmar with pro-Premium application (Hermex, Lublin, Poland) calculated the average daily TRP intake. The patients applied the balanced diet with a total caloric value of 2000 kcal and a daily intake of 50 g of protein, 270 g of carbohydrates, 70 g of fats, and 30 g of fiber. Everyone was administered a diet the day before the examination, with the TRP content calculated earlier. The content of TRP in food products was determined according to the findings of the Polish National Institute of Public Health (Warsaw, Poland). In Group IIA, the low FODMAP diet compared to the existing tryptophan content in the control group was recommended for 12 weeks. The patients in Group IIB were instructed to adopt a low FODMAP diet with L-tryptophan supplementation in a daily dose of 500 mg two times each at 8 a.m. and 8 p.m. for 12 weeks. Efforts were made not to change eating habits, but dietary consumption of tryptophan was controlled and kept constant. The optimal amount of protein, carbohydrates, fats, and fibers was maintained. The use of any drugs was forbidden, except for occasionally used laxatives. Patients from both groups were recommended to complete a diet diary daily under the control of competent dietitians, with whom they had telephone and e-mail contact. Dietary instruction was also provided to patients’ families and caregivers. After each week, the amount of TRP intake was analyzed to evaluate compliance with the recommendations. Follow-up medical examinations with the assessment of the symptoms and laboratory tests were performed after 12 weeks. This research was conducted as an open-label clinical trial. Written consent was obtained from all participants.

This study was conducted according to the guidelines of the Declaration of Helsinki and the Guidelines for Good Clinical Practice and approved by the Bioethics Committee of the Medical University of Lodz (RNN/176/18/KE); consent from the Commission was given on 15 May 2018.

### 2.4. Data Analysis

Our approach in the manuscript was to implement both parametric and non-parametric statistical tests. We chose the most appropriate test based on a thorough analysis of the variable’s consistency with a normal distribution. This approach allowed us to ensure the accuracy and reliability of our results. The normality of data distribution was checked using the Shapiro–Wilk W test. To analyze the differences between the two paired groups, we used Student’s *t*-test and the Wilcoxon matched-pairs signed-rank test, whereas, for the two unpaired groups, we used the Mann–Whitney U test. Simple linear regression was performed using the Spearman correlation with the rho rank correlation coefficient (t). Differences within groups before and after treatment were analyzed using the Wilcoxon signed-rank test. Differences were considered significant at *p* < 0.05. All statistical analyses were performed with STATISTICA 13.3 software (TIBCO Software Inc., Palo Alto, CA, USA).

## 3. Results

Both groups had similar characteristics and blood biochemical parameters, except for GFR. However, it is important to note that the GFR values in both groups were within the normal range, and they had no effect on the results of TRP metabolites expressed in mg per gram of creatinine This suggests that there is no significant difference between the two groups regarding overall health status (Table 1).

Tryptophan intake before treatment was 1209 ± 128 mg per day in the control group and 1200 ± 73 mg per day in the study group (*p* > 0.05). This tryptophan content in the diet was maintained in the second stage of this study.

There are no differences between groups in the concentration of TRP in urine. However, in the group of patients, the concentration of 5-HIAA was lower (*p <* 0.05) and the concentration of KYN and 3-IS was higher (*p <* 0.001, Table 2) compared with the control group.

**Figure 1:**
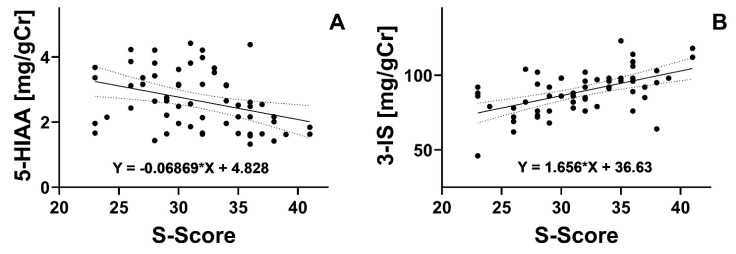
(**A**). Correlation between the severity of symptoms (S-score, points) and 5-hydroxyindoleacetic acid (5-HIAA, mg/gCr) in urine; *r =* −0.5266). (**B**). Correlation between symptom severity and urinary levels of 3-indoxyl sulfate (3-IS, _g/gCr); *r* = 0.3616. The correlations were analyzed with the Spearman rank test with the rho rank correlation coefficient (r).

A negative correlation was found between the severity of symptoms and concentration of 5-HIAA in urine (*p <* 0.001). However, the correlation between symptom severity and 3-IS concentration was positive (*p* < 0.01, Figure 1). No correlations were observed for urinary TRP and KYN excretion *(p* > 0.05).

**Figure 2:**
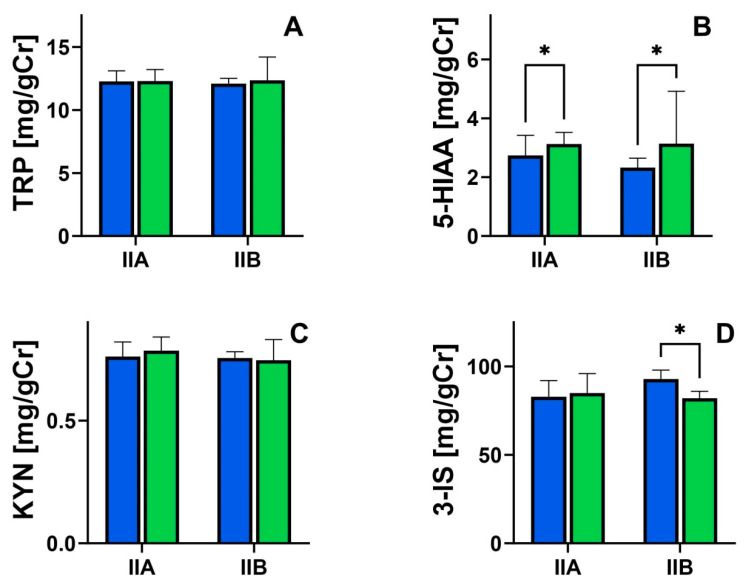
Urine concentration of tryptophan (TRP; (**A**)), 5-hydroxyindoleacetic acid (5-HIAA; (**B**)), kynurenine (KYN; (**C**)), indican (3-IS; (**D**)), and severity of symptoms in patients with functional constipation before (dark blue) and after nutritional treatment (green); the Wilcoxon matched-pair signed-rank test was used to compare before and after groups; * p < 0.05.

After nutritional treatment, urinary TRP excretion did not differ significantly in both groups (Figure 2A, *p* > 0.05). Similarly, no changes in kynurenine excretion were found (Figure 2C, *p* > 0.05). Nutritional treatment increased urinary 5-HIAA excretion in both study groups. In Group IIA, 2.82 ± 1.21 mg/gCr vs. 3.24 ± 0.92 mg/gCr (Figure 2B, *p* < 0.05), and in Group IIB, 2.92 ± 1.16 mg/gCr vs. 3.81 ± 1.38 mg/gCr (Figure 2B, *p* < 0.05). However, idican excretion decreased in Group IIB, i.e., in patients supplemented with L-tryptophan; 80.5 ± 13.8 mg/gCr vs. 74.9 ± 11.4 mg/gCr (Figure 2D, *p <* 0.05).

**Figure 3:**
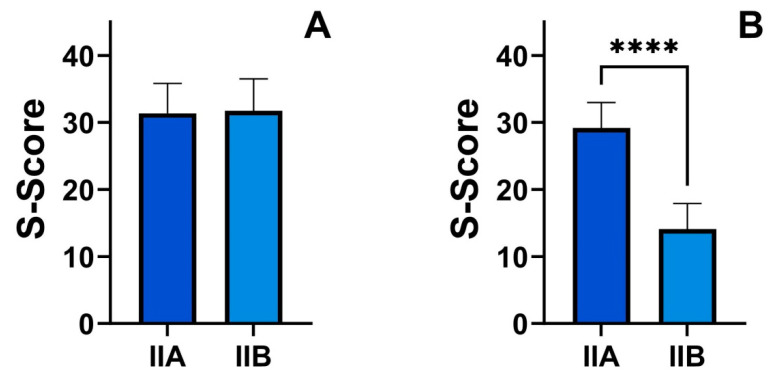
Comparison of symptom severity (S-score) in Group IIA (dark blue) and IIB (light blue) before (**A**) and after (**B**) nutritional intervention; differences in both groups before and after treatment were evaluated by the Wilcoxon signed-rank test, **** *p* < 0.001.

In a comparison of the results achieved after treatment in both groups, 5-HIAA levels in urine increased, and the intensity of symptoms decreased (Figure 3). However, the kynurenine and indole pathway indicators did not change (Table 3).

Patients were willing to follow the low FODMAP diet and cooperated well with the dietitians. L-tryptophan was well tolerated; only four (13.3%) patients reported increased fatigue in the afternoon during the first two weeks of the treatment without the need for discontinuation of the therapy or dose reduction. Additionally, 19 (63.3%) patients using L-tryptophan reported significantly improved sleep quality and duration.

## 4. Discussion

Treating chronic constipation and other functional diseases of GIT in older people is a difficult task. The use of pharmacological drugs is limited due to their side effects, especially in patients with comorbidities. In recent decades, a low FODMAP diet has often been recommended for the treatment of IBS [22,23,24], and numerous studies have demonstrated its effectiveness in most patients, especially those with IBS-D [22,23,24,25,26]. The results of IBC-C treatment were weaker and required modification [27,28], such as the simultaneous administration of probiotics, which can beneficially alter the gut microbiome and relieve abdominal symptoms [29,30,31,32]. Some probiotics may improve the stool consistency and frequency of bowel movements [33,34,35,36]. It was considered that the gut microbiome can predict low FODMAP diet efficacy [37]. On the other hand, it was shown that adding probiotics to a low FODMAP diet does not contribute to symptom response [38]. It was also shown the effects of a low FODMAP diet on the colonic microbiome appear only to Bifidobacterium with no consistent impact on the other bacteria [39]. On the contrary, other studies showed reduced Bifidobacterium in patients with dysbiosis [40].

Changes in gut microbiota occur under the influence of various factors, including food ingredients, which are a substrate for the synthesis of many biologically active compounds, including TRP [41,42]. Our results indicate that one of the causes of chronic constipation in older people may be changes in TRP metabolism. In particular, the activity of the serotonin pathway was reduced, which is reflected in a lower level of 5-HIAA in urine compared to the control group. These changes may be due to several reasons. One may be a lower activity of tryptophan-metabolizing enzymes in some people at this age. At the same time, the activity of the kynurenine and indole pathways was increased. Intestinal bacteria significantly influence the activity of both metabolic pathways. Bacterial antigens and pro-inflammatory cytokines increase the expression of IDO-1, which has a greater affinity to TRP than TPH-1 [14,15].

High activity of the indole pathway, which results in an increased level of indican (3-indole sulfate) in urine, may have an important impact on the functional disorders of the GIT. Indican is synthesized via tryptophanase-expressing colon anaerobic bacteria on the pathway in the following order: L-tryptophan; indole; and indoxyl-3-indole sulfate. Indican is considered a quantitative biomarker of the state of the intestinal microbiome [43]. However, this TRP metabolic pathway is mainly metabolized by the enzymes of anaerobic bacteria in the large intestine. Under their influence, many biological compounds are formed, including methane and short-chain fatty acids (SCFAs) [44,45].

Methane is generated by bacteria and archaea through anaerobic fermentation of ingested polysaccharides [46]. It can affect 5-HT concentration in the colon mucosa and reduce the motor activity of this organ [47,48,49]. Nevertheless, high methane production does not always correlate with constipation [50] and occurs in healthy people [51].

Short-chain fatty acids (SCFAs), including indoleacetic acid (ILA), indole propionic acid (IPA), and butyric acid (EAA), regulate mucosal blood flow, fluid and electrolyte balance, and intestinal motility [52,53]. These metabolites can contribute to constipation and lead to diarrhea if their concentration is too high [54,55]. Excitation or inhibition of gut motility depends on the levels of these metabolites [56,57,58,59].

Gut microbiota generally regulate intestinal functions by participating in TRP metabolism because their metabolites are involved in smooth muscle contraction or relaxation through different 5-HT receptors. The growth of intestinal bacteria might lead to the upregulation of the SERT expression as a result of a decrease in 5-HT concentration in colon mucosa and a decrease in the motor activity of the colon [60]. On the contrary, the same researchers reported that increased 5-HT concentrations in the blood or colonic mucosa might be related to constipation [61,62]. These differences may also be due to the balance between 5-HT receptor activation and desensitization [19].

The contribution of the composition of intestinal bacteria to these complex processes is also essential. It differs between constipated patients and healthy people, and alternations were associated with constipation [63,64]. Microbiota regulates 5-HT production via several mechanisms [65]. It is suggested that gut bacteria may also upregulate the production of kynurenines and indole from TRP, thereby reducing the substrate for the production of 5-HT [66]. Such a mechanism is possible in our patients, but dysbiosis’s involvement in constipation’s pathogenesis via regulating SERT activity is also possible. In addition, some bacteria may directly stimulate the production of serotonin. Fecal microbiota transplantation from healthy donors has a beneficial effect on patients suffering from diarrhea and constipation, confirming the complexity of the impact of the gut microbiome on functions of the digestive tract [67]. The impact of nutritional ingredients is likely complex. Each diet should also be personalized and adapted to the patient’s health condition. Our results show that in functional constipation, it is advisable to increase the intake of tryptophan in the diet. Nevertheless, increasing the tryptophan content is usually associated with increased caloric value of meals, which some patients do not accept. An alternative method is TRP supplementation. Increasing TRP intake above 2000 mg per day reduced constipation and was well tolerated by patients. TRP released from the capsule in the intestines has easy access to the enzymes synthesizing 5-HT. Tryptophan supplementation was mainly used in patients with sleep disorders and depression. For this purpose, L-tryptophan doses of 0.5 to 5.0 g have been recommended. Side effects such as nausea, dizziness, and headaches rarely occur in some patients [68].

The involvement of 5-HT in the pathogenesis of these disorders is sufficiently documented. However, the role of other pathways and metabolites is still poorly understood. Kynurenines may have beneficial and unfavorable effects on the central and visceral nervous system, which, among other factors, depend on their levels. The properties of indole metabolites are also little known. It is generally accepted that most of them have anti-inflammatory and immuno-regulatory properties. However, indican may be neurotoxic, especially in high concentrations. Changes in the level of the above metabolites may significantly contribute to the pathogenesis of gastrointestinal diseases. They are caused by many factors, including gut microbiota, playing an essential role in regulating the functions of the gut–brain axis. The results confirmed the opinions of many researchers who recognize the important role of diet and microbiome in the pathogenesis of functional gastrointestinal diseases by regulating TRP metabolism [69,70]. Nevertheless, further research is necessary, especially in determining the strains of bacteria involved in these processes. So far, it has not been precisely established which probiotics and in what doses have therapeutic properties. The length of therapy is also under discussion. Some bacteria can colonize the intestines; others are tourists. It is necessary to standardize research methods assessing the microbiome status, which is not constant but can change over time. Simple methods are needed to monitor these changes. Indirect tests, such as hydrogen–methane breath tests and testing of bacterial metabolites in urine, are helpful for this purpose. However, for cognitive purposes, genetic testing is necessary. It can be assumed that the researcher’s great interest in these problems will bring further results of scientific and clinical value.

In summary, the pathogenesis of functional gastrointestinal diseases is complex, but 5-HT homeostasis disorders play a crucial role. Serotonin levels in the intestinal mucosa and the blood depend on the expression of tryptophan hydroxylase and the activity of selective 5-HT reuptake transporters [71]. The level of their expression is influenced by nutritional and bacterial factors [17]. However, the main condition is consuming an appropriate amount of substrate for their synthesis, i.e., exogenous L-tryptophan. For this reason, diet is still a main element of the treatment of gastrointestinal diseases.

Our study has some limitations. The group of respondents is relatively small. This study was conducted as an open-label clinical trial without a placebo. The severity of FC symptoms is based mainly on patients’ subjective assessment. These criteria apply to all functional diseases of GIT. Nevertheless, the assessment of nutrition quality also depends on patients’ complaints, but good cooperation with professional dietitians ensures the reliability of the results.

## 5. Conclusions

A low FODMAP diet supplemented with L-tryptophan benefits elderly patients with functional constipation. The changes induced by that diet concern the increase in 5-HT production. The increased intake of TRP may influence its level and the activity and balance of its metabolic pathways involving the gut microbiome. Further research is necessary, especially on the type of bacteria involved in TRP metabolism.

## Figures and Tables

**Table 1:** General characteristics and the selected biochemical blood parameters in older patients without abdominal complaints (Group I) and with functional constipation (Group II); average ± SD.

Feature	Group I (*n* = 40)	Group II (*n* = 60)	*p*
Age (years)	72.1 ± 8.6	73.3 ± 10.9	0.56
Gender M/F	9/31	15/45	-
BMI (kg/m^2^)	23.4 ± 2.1	23.9 ± 1.8	0.31
GFR (ml/min)	95.8 ± 4.9	92.1 ± 7.7	0.008
ALT (U/L)	18.6 ± 3.9	17.8 ± 4.6	0.37
AST (U/L)	16.2 ± 2.3	17.2 ± 2.6	0.052
CRP (mg/L)	3.8 ± 1.6	4.3 ± 2.8	0.31
FC (µg/g)	32.3 ± 19.8	36.9 ± 18.3	0.24

M—male, F—female, BMI—body mass index, GFR—glomerular filtration rate, ALT—alanine aminotransferase, AST—asparagine aminotransferase, CRP—C-reactive protein; differences between groups were evaluated by the Mann–Whitney U test.

**Table 2:** Comparison of results between controls (Group I) and patients with functional constipation (Group II) of urinary excretion of tryptophan (TRP) and its metabolites: 5-hydroxyindoleacetic acid (5-HIAA), kynurenine (KYN), and indican (3-IS); all results are expressed in mg/gCr; mean ± SD; differences between both groups were assessed by the Mann–Whitney U test.

Lab Test	Group I (*n* = 40)	Group II (*n* = 60)	*p*-Value
TRP	13.7 ± 1.91	13.9 ± 12.1	>0.05
5-HIAA	3.68 ± 0.71	3.34 ± 1.21	<0.05
KYN	0.46 ± 0.12	0.76 ± 0.13	<0.001
3-IS	35.2 ± 12.2	80.1 ± 12.9	<0.001

**Table 3:** Urinary excretion of tryptophan (TRP) and its metabolites: 5-hydroxyindoleacetic acid (5-HIAA), kynurenine (KYN), 3-indoxyl sulfate (3-IS), and severity of symptoms (S-score, points) in patients with functional constipation after nutritional treatment; all laboratory results expressed in mg/gCr; average ± SD; differences in both groups before and after treatment were evaluated by the Wilcoxon signed-rank test.

Feature	Group IIa	Group IIb	*p*-Value
TRP (mg/gCr)	11.9 ± 2.93	15.1 ± 14.9	>0.05
5-HIAA (mg/gCr)	3.04 ± 0.92	3.81 ± 1.38	<0.05
KYN (mg/gCr)	0.97 ± 0.19	0.95 ± 0.24	>0.05
3-IS (mg/gCr	80.5 ± 13.8	74.9 ± 11.4	>0.05
S-score (points)	29.2 ± 3.86	14.3 ± 3.81	<0.001

## Data Availability

The data supporting the reported results can be provided by the corresponding author.

## Institutional Board Statement

The study was approved by the Bioethics Committee of the Medical University of Lodz (RNN/176/18/KE); consent from the Commission was given on 15 May 2018.
